# Prevalence and correlates of carotid artery stenosis in a cohort of Sri Lankan ischaemic stroke patients

**DOI:** 10.1186/s12883-021-02415-1

**Published:** 2021-10-04

**Authors:** K. C. D. Mettananda, M. D. P. Eshani, L. M. Wettasinghe, S. Somaratne, Y. P. Nanayakkkara, W. Sathkorala, A. Upasena, C. Sirigampola, P. M. Y. Tilakaratna, A. Pathmeswaran, U. K. Ranawaka

**Affiliations:** 1 Department of Pharmacology, Faculty of Medicine, University of Kelaniya, Talagolla Road, Ragama, Sri Lanka; 2 Stroke Unit, Colombo North Teaching Hospital, Ragama, Sri Lanka; 3 Department of Medicine, Faculty of Medicine, University of Kelaniya, Ragama, Sri Lanka; 4 Radiology Unit, North Colombo Teaching Hospital, Ragama, Sri Lanka; 5 University Medical Unit, North Colombo Teaching Hospital, Ragama, Sri Lanka; 6 Department of Public Health, Faculty of Medicine, University of Kelaniya, Ragama, Sri Lanka

**Keywords:** Carotid stenosis, South Asians, Ischemic strokes, Prevalence, Risk-factors, Sri Lanka

## Abstract

**Background:**

Large artery atherosclerotic disease is an important cause of stroke, accounting for 15–46% of ischaemic strokes in population-based studies. Therefore, current guidelines from west recommend urgent carotid imaging in all ischaemic strokes or transient ischaemic attacks and referral for carotid endarterectomy. However, the clinical features and epidemiology of stroke in Asians are different from those in Caucasians and therefore the applicability of these recommendations to Asians is controversial. Data on the prevalence of carotid artery stenosis (CAS) among South Asian stroke patients is limited. Therefore, we sought to determine the prevalence and associated factors of significant CAS in a cohort of Sri Lankan patients with ischaemic stroke.

**Methods:**

We prospectively studied all ischaemic stroke patients who underwent carotid doppler ultrasonography admitted to the stroke unit of a Sri Lankan tertiary care hospital over 5 years. We defined carotid stenosis as low (< 50%), moderate (50–69%) or severe (70–99%) or total-occlusion (100%) by North American Symptomatic Trial Collaborators (NASCET) criteria. We identified the factors associated with CAS ≥ 50% and ≥ 70% by stepwise multiple logistic regression analysis.

**Results:**

A total of 550 ischaemic stroke patients (326 (59.3%) male, mean age was 58.9 ± 10.2 years) had carotid doppler ultrasonography. Of them, 528 (96.0%) had low-grade, 12 (2.2%) moderate and 7 (1.3%) severe stenosis and 3 (0.5%) had total occlusion. On multivariate logistic regression, age was associated with CAS ≥ 50% (OR 1.12, *p* = 0.001) and CAS ≥ 70% (OR 1.14, *p* = 0.016), but none of the other vascular risk factors studied (sex, hypertension, diabetes mellitus, smoking, past history of TIA, stroke or ischemic heart disease) showed significant associations.

**Conclusions:**

Carotid stenosis is a minor cause of ischemic stroke in Sri Lankans compared to western populations with only 4.0% having CAS ≥ 50 and 3.5% eligible for carotid endarterectomy. Our findings have implications for the management of acute strokes in Sri Lanka.

## Background

Large artery atherosclerotic disease is an important cause of stroke, accounting for 15–46% of ischaemic strokes in population-based studies [1–5], and is considered the stroke subtype with the highest recurrence risk [6, 7]. Extracranial internal carotid artery stenosis (CAS) is the most important cause of large artery stroke [1]. Carotid endarterectomy (CEA) has been shown to reduce the relative risk of disabling stroke or death by 48% in patients with severe CAS [8–11] and by 27% in patients with moderate CAS [8, 9, 12, 13]. Latest acute stroke management guidelines recommend referring all patients with ischaemic strokes/transient ischaemic attacks who are candidates for carotid intervention, for carotid imaging as soon as possible, preferably within 24 h [14–17]. However, the applicability of these recommendations in different populations depends on the local prevalence of CAS and their risk associations. Carotid artery stenosis (CAS) is known to be more associated with White patients and is less among Black or Asian patients [18]. However, data on the prevalence of CAS and its risk associations among South Asian stroke patients is limited. Therefore, we sought to study the prevalence and correlates of CAS in a cohort of Sri Lankan ischaemic stroke patients.

## Methods

We prospectively studied all ischaemic stroke patients admitted to the stroke unit of the Colombo North Teaching Hospital (CNTH), Sri Lanka over 5 years (2014–2019); The Ragama stroke registry. The CNTH is a 1550-bed tertiary care hospital situated in the suburban city of Ragama, 18 km from the capital city of Colombo. CNTH is the only tertiary care hospital in the administrative district of Gampaha. Sri Lanka is divided into 25 administrative districts and Gampaha is the second-most populated district in the country, with 11.32% of the total population and a population density of 1711/km2. Gampaha has the whole spectrum of rural, semi-urban and urban populations. The Stroke Unit at the CNTH is the only stroke unit in the Gampaha district.

Data on demographics, vascular risk factors, stroke characteristics and outcome were recorded prospectively by trained investigators using an interviewer-administered questionnaire, supplemented by medical records. All ischemic stroke patients who underwent Carotid doppler ultrasonography were included in this study.

Carotid stenosis was defined as low (< 50%), moderate (50–69%) or severe (70–99%) or total-occlusion (100%) by the percentage stenosis according to North American Symptomatic Carotid Endarterectomy Trial (NASCET) classification [10].

A total of 891 stroke patients were admitted to the stroke unit over the 5 years, and 765 (85.5%) of them were ischaemic strokes. Of all patients with ischaemic strokes, 550 (71.9%) had undergone carotid doppler studies as in-patients and were recruited for this study.

Data were analysed using IBM SPSS statistics version 22.0. Continuous variables were reported as means with standard deviation (SD), and categorical variables were reported as percentages. The significance level was set at *p* < 0.05. Factors associated with CAS ≥ 50% and ≥ 70% were separately identified by stepwise multiple logistic regression analysis.

Ethical approval was obtained from the Ethics Review Committee of the Faculty of Medicine, University of Kelaniya, Sri Lanka. Informed written consent of the patients was obtained.

## Results

A total of 550 patients with ischaemic stroke who underwent carotid doppler studies as in-patients at the stroke unit over the 5 year study period formed the study population. There were 326 (59.3%) males, with a mean age of 58.9 ± 10.2 years. All have had brain CT or MRI scans done. Baseline characteristics and vascular risk factors of the study population are shown in Table 1.

**Table 1:** Baseline characteristics and vascular risk factor profiles of the study population

	Frequency (%) (*n* = 550)
**Demographic characteristics**	
Male sex	326 (59.3)
Age > 60 years	293 (53.3)
Ethnicity	
Sinhalese	517 (94.0)
Tamil	14 (2.5)
Muslim	13 (2.4)
Burgher	5 (0.9)
Chinese	1 (0.2)
**Vascular risk factors**	
Hypertension	347 (63.1)
Diabetes mellitus	307 (55.8)
Hyperlipidaemia	169 (30.7)
Current smoking	137 (24.9)
Previous IHD^a^	99 (18.0)
Previous stroke	94 (17.1)
Previous TIA^b^	48 (8.7)

^a^*IHD* ischaemic heart disease

^b^*TIA* transient ischaemic attacks

Prevalence of CAS; 528 (96.0%) had low degree (< 50%) stenosis, 12 (2.2%) had moderate (50–69%) stenosis, 7 (1.3%) had severe (70–99%) stenosis, and 3 (0.5%) had total occlusion of the extracranial internal carotid artery on the symptomatic side. CAS of ≥50% was seen in 22 (4.0%) patients.

Associations of both CAS ≥ 50% and CAS ≥ 70% on stepwise multiple logistic regression analysis are shown in Table 2. Age was independently associated with both CAS ≥ 50% (OR 1.12, *p* = 0.001) and CAS ≥ 70% (OR 1.14, *p* = 0.016). The other vascular risk factors studied, i.e. male sex, hypertension, diabetes mellitus, smoking, past history of TIA, stroke or ischemic heart disease were not significantly associated with CAS.

**Table 2:** Associations of vascular risk factors with different degrees of carotid artery stenosis

	< 70% CAS group *n* = 540	≥70% CAS group *n* = 10	Association with ≥70% CAS		< 50% CAS group *n* = 528	≥50% CAS group *n* = 22	Association with ≥50% CAS	
	n,(%)	n,(%)	OR	p*	n,(%)	n,(%)	OR	p*
Age (years)			1.14	0.016			1.12	0.001
Male sex	318(58.9)	8(80.0)	0.43	0.331	311(58.9)	15(68.2)	0.79	0.659
Hypertension	342(63.8)	5(50.0)	0.68	0.583	335(63.4)	12(54.5)	0.71	0.493
Diabetes mellitus	304(56.6)	3(30.0)	0.42	0.238	298(56.4)	9(40.9)	0.58	0.252
Hyperlipidaemia	164(30.8)	2(20.0)	0.84	0.849	193(36.8)	6(27.3)	0.88	0.826
Smoking	134(25.1)	3(30.0)	1.36	0.697	131(24.8)	3(27.3)	1.31	0.631
Previous TIA	48(9)	0(0)	0.00	0.997	47(8.9)	1(4.5)	0.61	0.643
Previous stroke	94(17.5)	0(0.0)	0.00	0.996	91(17.2)	3(13.6)	0.81	0.743
Previous IHD	96(17.8)	3(30.0)	2.47	0.239	95(18.0)	4(18.2)	1.06	0.921

*TIA* transient ischaemic attack, *IHD* ischaemic heart disease, *CAS* Carotid artery stenosis

^*^Adjusted for all the variables in the model

## Discussion

To our knowledge, this is the first study describing the prevalence and risk associations of CAS among Sri Lankan stroke patients. We observed low prevalence rates of significant carotid artery stenosis, with CAS ≥ 50% in only 4.0%, 70–99% in only 1.3% and total occlusion in 0.5%. This finding has major implications in adopting the UK, European or American Stroke guidelines to Sri Lanka and possibly to South Asia.

Ethnic differences in stroke aetiologies are well recognised [19–22]. While western countries have more cardioembolic strokes (20–35%), Asians have more atherosclerotic strokes (25–65%). Prevalence of small-vessel disease is higher in Asians (prevalence ~ 50%) than in Caucasians (prevalence ~ 20%) [23]. Embolic strokes due to emboli originating from the heart or extracranial large arteries are common in Western populations, whereas small-vessel occlusion or intracranial atherosclerosis is more prevalent in Asians [23]. Whites have more extracranial carotid atherosclerosis [20, 24], whereas Asians have more intracranial atherosclerotic disease [22, 25, 26]; 54% of ethnic South Asians living in Singapore [27] and 29.4% of Indian patients in the Hyderabad stroke registry [28] were reported to have intracranial large-artery disease. Among ischaemic stroke subtypes, lacunar strokes, which were once the commonest variety in Asians, are now declining. Emerging data suggest that large artery atherosclerosis and in particular that of intracranial vessels is the predominant aetiology in most Asian countries [21].

Studies of multi-ethnic populations have shown that whites have a higher prevalence of CAS than non-whites [18, 24, 29–33]. Community prevalence of CAS in the USA is reported to be highest among native Americans (OR 1.3) and lowest in African Americans (OR 0.69) and Asians (OR 0.65) compared to Caucasians [32]. Prevalences of CAS ≥ 80% in a USA hospital-based study were, 9.2% in whites compared to 4.3% in Hispanics, 2.9% in blacks, and 2.8% in Asians [34]. Similarly, the prevalence of CAS ≥ 50% in a multi-ethnic population in the Netherlands, were 16% among whites, 10% among blacks and 9% among Asians and the adjusted odds ratio of having CAS ≥ 50% in non-white populations compared to white populations was 0.44 [18]. Carotid atherosclerosis in a population-based study of Canada reported higher among Europeans (0.75 mm) than in South Asians (0.72 mm) and Chinese (0.69 mm) [31].

Low rates of extracranial CAS in Asian populations could be related to several reasons. The differential prevalence of vascular risk factors observed between Asian and white Caucasian populations is one. Risk factors known to be associated with extracranial CAS such as older age, smoking and hyperlipidaemia [35–37] are commoner among western populations [38–40], whereas factors associated with intracranial atherosclerosis such as diabetes mellitus and metabolic syndrome [35–37, 40] are more prevalent in Asians [41, 42]. Comparing our study with a contemporary study from the UK, our study had lower extracranial CAS > 50% (4.0% vs 7.9%), patients were younger (mean age 58.9 vs 71.4 years) and had lower rates of smoking (25% vs 35%) and hyperlipidaemia (30% vs 55%) and higher prevalence of diabetes mellitus (56% vs 24%), mostly leading to a vascular phenotype favouring intracranial atherosclerosis [33].

Prevalence of severe CAS among our population (1.8%) was even lower when compared with recent data from other South Asian countries. CAS ≥ 70% is reported in 10% of stroke patients from India [43] and 12% from Pakistan [44]. The different prevalence rates may be partly due to methodological differences between studies. Compared to our study (*n* = 550), the study by Bharathykunisetty [43] was only 100 patients recruited over a one-year duration. We studied only extracranial internal carotid artery stenosis, but some studies have reported both extracranial and intracranial atherosclerosis which would naturally yield a higher rate of stenosis; e.g. Razzaq et al. reported carotid atherosclerosis in 22% of young stroke patients from Pakistan, but extracranial carotid stenosis was seen in only 7.9% [45]. Furthermore, we studied only symptomatic stenoses, but some studies have included both symptomatic and asymptomatic stenoses in their analyses, e.g., Wasay et al. reported CAS of ≥70% in 12% of patients but only 7% were symptomatic [44]. Compared to older studies from South Asia, better medical care and risk factor management may have contributed to the low prevalence rate in our study. Sri Lanka is considered a role model in health development in a developing country and has consistently performed better than its South Asian neighbours in terms of health and social indicators, such as the Human Development Index, life expectancy at birth and female literacy rate [46]. It is, therefore, plausible that lower prevalence rates seen in our study compared to other South Asian countries may also be related to better vascular risk factor management in Sri Lanka.

We observed that significant CAS was associated with increasing age but not with sex or other common vascular risk factors (hypertension, diabetes, hyperlipidaemia, smoking, previous TIA/stroke). Tan et al. reported similar findings in their study of Taiwanese patients [26]. Other studies have noted CAS to be associated with risk factors such as male sex, smoking, diabetes mellitus, hypertension, hypercholesterolemia and past history of stroke [33, 35, 43, 47], but there is much variation in the associations reported between studies.

Strengths of our study include the large number of consecutive patients with ischaemic stroke studied in a single unit over a long period of 5 years, confirmation of ischaemic stroke with CT and/or MRI scanning in all patients, and prospective data collection minimizing recall bias and selection bias. However, some limitations need to be acknowledged. Only 72% of patients admitted with ischaemic stroke during the study period had carotid doppler assessments. Due to resource limitations, carotid doppler studies were not done in all patients during the hospital stay. Further, patients who were too ill to be transported to the Radiology department did not have doppler scanning. We have included data from all patients who had carotid doppler studies during the hospital stay.

However, our carotid imaging rate was much higher compared to in some Asian studies (38%) [48], and comparable to some studies from more developed countries such as the USA (79%) [24]. As the study was conducted over 5 years, carotid doppler studies were done by three different radiologists with more than 10 years’ experience, and therefore, some inter-observer variability in the assessment of the exact percentage of CAS is a possibility. However, since the patients were categorised into only 4 categories, < 50%, 50–69%, 70–99, 100% the effect of this on final occlusions would be minimal. Our data are from hospital admissions in a single tertiary care centre and therefore may not be generalizable to the community at large. Furthermore, we reported only the prevalence of extracranial CAS, but the burden of undetected intracranial atherosclerosis is not reflected in our study.

## Conclusions

We report the first data on CAS in Sri Lankan patients with ischaemic stroke. Clinically significant CAS of ≥50% and 70–99% is seen among 4.0 and 1.3% of Sri Lankan ischaemic stroke patients, respectively. These figures are much lower compared to Western data, and further research is needed to understand the reasons behind the low prevalence rates in our patients. Our findings have implications in the uptake of western guidelines on acute stroke management in Sri Lankan stroke patients. Cost-effectiveness in urgent carotid imaging in all patients with TIA or stroke is questionable in Sri Lankans including South Asians and probably we need to adopt western guidelines with suitable amendments to suit local disease epidemiology.

## Data Availability

The datasets used and/or analysed during the current study are available from the corresponding author on reasonable request.

## Abbreviations

CAS: Carotid artery stenosis
NASCET: North American Symptomatic Trial Collaborators
CEA: Carotid endarterectomy
CNTH: Colombo North Teaching Hospital
